# Supplementary material for: Methods to achieve near-millisecond energy relaxation and dephasing times for a superconducting transmon qubit
Source: Nat Commun. 2025 Jul 8;16:5421. doi: 10.1038/s41467-025-61126-0 (PMC12238396; doi:10.1038/s41467-025-61126-0)
Supplement: Supplementary file 1 — Supplementary Information [file 41467_2025_61126_MOESM1_ESM.pdf]

SUPPLEMENTARY INFORMATION FOR "METHODS TO ACHIEVE NEAR-MILLISECOND ENERGY RELAXATION AND DEPHASING TIMES FOR A SUPERCONDUCTING TRANSMON QUBIT"

Mikko Tuokkola[1,*], Yoshiki Sunada[1], Heidi Kivijärvi[1], Jonatan Albanese[1], Leif Grönberg[2], Jukka-Pekka Kaikkonen[2], Visa Vesterinen[2], Joonas Govenius[2], and Mikko Möttönen[1,2,†]

[1]QCD Labs, QTF Centre of Excellence, Department of Applied Physics, Aalto University, P.O. Box 13500, FIN-00076 Aalto, Finland
[2]VTT Technical Research Centre of Finland Ltd. & QTF Centre of Excellence, P.O. Box 1000, 02044 VTT, Finland

[*]Corresponding author email: mikko.tuokkola@aalto.fi
[†]Corresponding author email: mikko.mottonen@aalto.fi

SUPPLEMENTARY NOTE 1: SUPPLEMENTARY DATA FIGURES AND TABLES FOR THE MAIN TEXT

Supplementary Table 1. Comparison of high-coherence transmon qubits in the existing literature and this work. Qubit frequency f_q, median or average energy relaxation time T_1, the corresponding quality factor $Q := 2\pi f_\mathrm{q} T_1$, and median or average echo dephasing time T_2^echo are shown.

Main text Ref.	f_q (GHz)	T_1 (µs)	Q ($\times 10^6$)	T_2^echo (µs)
9	3.73	303	7.1	201
10	3.92	401	9.9	N/A
11	3.86	393	9.5	N/A
12	4.24	292	7.7	N/A
13	5.92	280	10.4	238
14	3.02	270	5.1	307
15	3.95	323	8.0	286
16	4.79	210	6.5	290
This work	2.86	425	7.7[a]	541

[a] Qubit frequency in the relaxation time measurement is 2.89 GHz.

Supplementary Table 2. Equipment used in the fabrication process.

Equipment	Description
Sputtering system	Eclipse Mark IV
Maskless aligner	Heidelberg Instruments MLA150
Plasma processing system	Oxford Instruments Plasmalab 80 Plus
Electron-beam writer	Raith EBPG5200
Electron-beam evaporator	Plassys MEB700S2-III
Vapor prime oven	YES III
Dicing saw	DISCO DFD6561

Supplementary Table 3. Equipment used in the measurement setup.

Equipment	Description	Abbreviation
Dilution refrigerator	Bluefors XLD400sl	
RFSoC evaluation board	Xilinx Zynq UltraScale+ RFSoC ZCU216 with add-ons CLK104 and XM655	
Frequency standard	Stanford Research Systems FS725 10-MHz Rb Standard	
Coaxial cables	Totoku TCF258AA2000/1500 (room temperature)<br>Mini-Circuits 141-72SM+ (room temperature)<br>Bluefors 0.86 mm SCuNi-CuNi semi-rigid (cryogenic input lines)<br>Bluefors 0.86 mm NbTi-NbTi semi-rigid (cryogenic output lines, MXC to 4 K)<br>Bluefors 2.19 mm SCuNi-CuNi semi-rigid (cryogenic output lines, 4 K to RT)	
HEMT amplifiers	Low Noise Factory LNF-LNR4_8F (room temperature)<br>Low Noise Factory LNF-LNC4_8F (cryogenic)	HEMT<br>HEMT*
Low-pass filters	RLC F-30-8000-R (3-dB point = 8.4 GHz, 60-dB stopband = 10.8–40 GHz)<br>Mini-Circuits VLF-5850+ (3-dB point = 6.54 GHz)<br>Aivon Therma uD25 pairwise RC-filter (1.5 kΩ per wire, 3-dB point = 1 kHz)	LPF<br>LPF*<br>LPF†
Bandpass filters	Mini-Circuits VBFZ-3590+ (2-dB passband = 3–4.3 GHz)<br>Mini-Circuits VBFZ-6260-S+ (2-dB passband = 5.6–7 GHz)	VBFZ<br>VBFZ*
Highpass filter	Mini-Circuits VHF-8400+ (20-dB stopband = DC–7.8 GHz)	HPF
Eccosorb filter	Kawashima Manufacturing 3.5mm Filter Connector L9.6 (0.32 dB/GHz)	Ecco.
Attenuator	Bluefors cryogenic attenuators<br>XMA 2082-6418-20-CRYO<br>Mini-Circuits BW-S10-2W263+ (room temperature)	X dB<br>20 dB*<br>10 dB†
Isolators	Low Noise Factory LNF-ISISC4_8A (4–8 GHz)<br>Low Noise Factory LNF-ISISC4_12A (4–12 GHz)	
Circulator	Low Noise Factory LNF-CIC4_12A (4–12 GHz)	
TWPA	Supplied by VTT (Technical Research Center of Finland Ltd.)	TWPA
TWPA pump generator	Valon 5015 Frequency Synthesizer	
Voltage source	Stanford Research Systems SIM928 Isolated Voltage Source	V
Sample holder	QDevil QCage.24 with QCage Magnetic Shielding	QCage.24

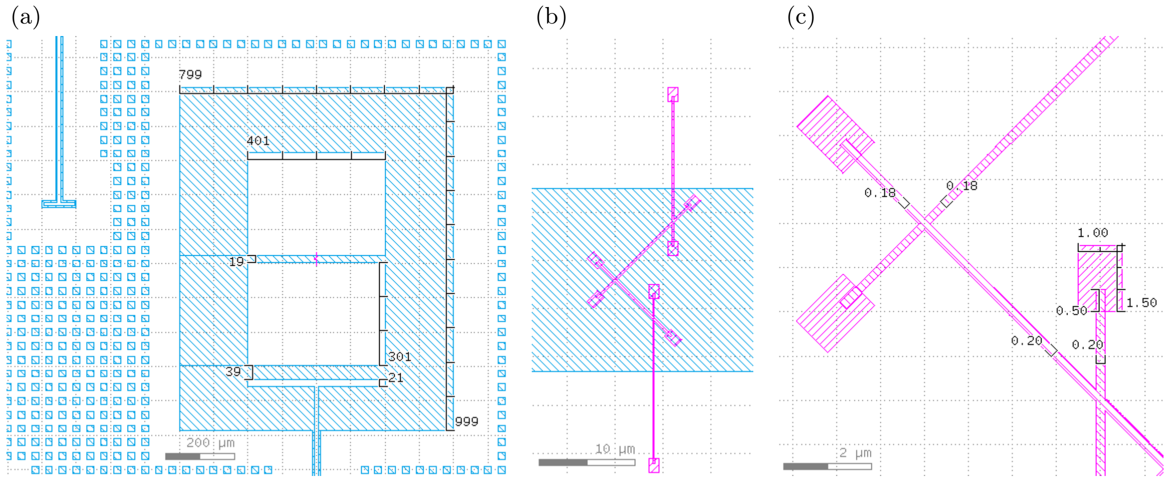

Supplementary Fig. 1. **Lithographic pattern of qubit $\mathbf{Q_2}$.** (a) Overview of our transmon qubit with its drive line (top left) and coupling capacitor of the readout resonator (bottom). The Nb film in the shaded region is removed by photolithography and dry etching. (b) Close-up of the pattern for the electron-beam lithography of the Josephson junction (diagonal strips) and the bandages for galvanically connecting it to the Nb capacitor (vertical strips). The rectangular parts at the tips of each strip are exposed at a lower dose than the other exposed regions and becomes an undercut in the two-layer resist stack. (c) Close-up of the Josephson junction. The dimensions of the Josephson junction are 180 nm by 180 nm in the mask pattern.

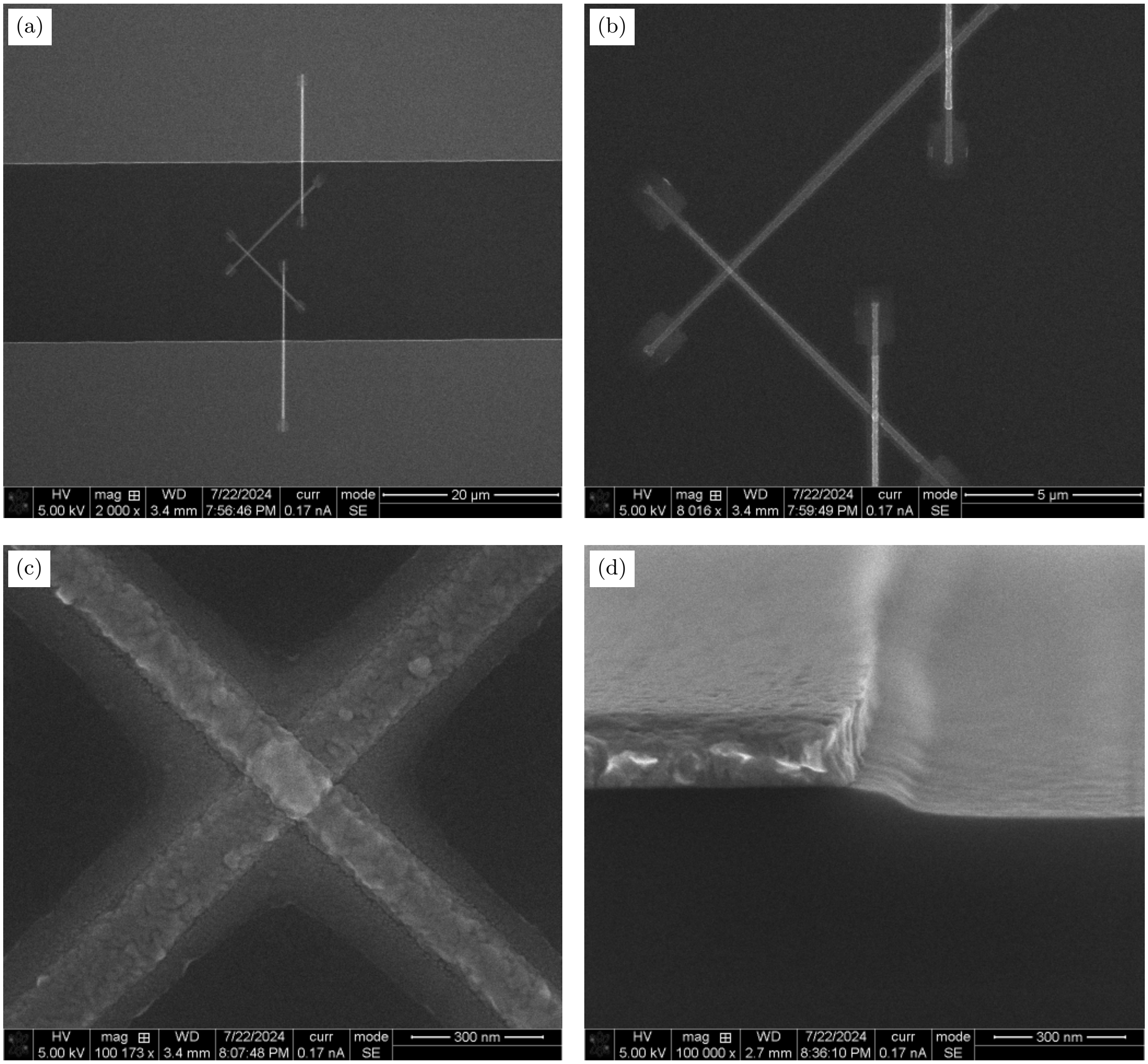

Supplementary Fig. 2. **Scanning-electron-microscope (SEM) images of a chip processed in the same fabrication run as the sample measured in this work.** (a) Josephson junction (diagonal strips) and the bandages (vertical strips) for galvanically connecting it to the Nb capacitor (top and bottom gray regions). (b) Close-up of the Josephson junction. (c) Further close-up of the Josephson junction. The junction shown here, which is fabricated using a 140 nm by 140 nm mask pattern, is measured to be approximately 170 nm by 140 nm. This implies that the junction of qubit Q_2 is likely approximately 210 nm by 180 nm. (d) Etch profile of the Nb film (left) on the Si substrate (bottom) obtained by cleaving the chip.

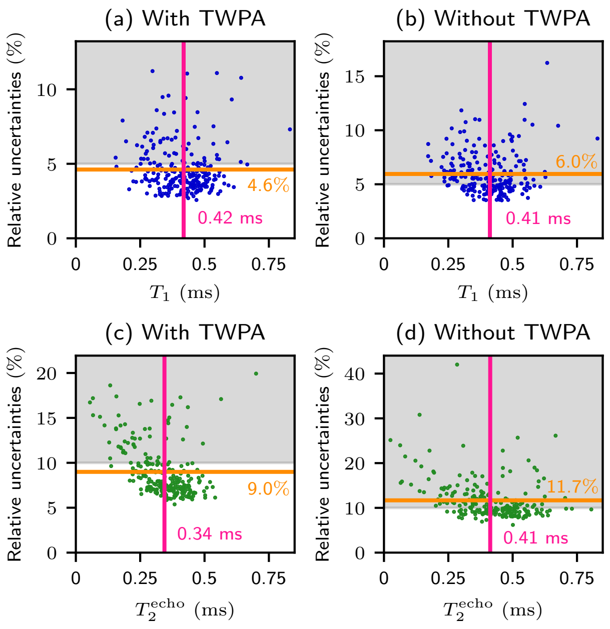

Supplementary Fig. 3. **Energy relaxation and echo dephasing times and their relative uncertainties for qubit Q_2 measured during the second cooldown.** (a) T_1 measured while pumping the TWPA. (b) T_1 measured without pumping the TWPA. (c) T_2^{echo} measured while pumping the TWPA. (d) T_2^{echo} measured without pumping the TWPA. The orange horizontal lines and the pink vertical lines represent the average uncertainties and the median values of T_1 or T_2^{echo}, respectively. The values in the grey area are excluded from further analysis.

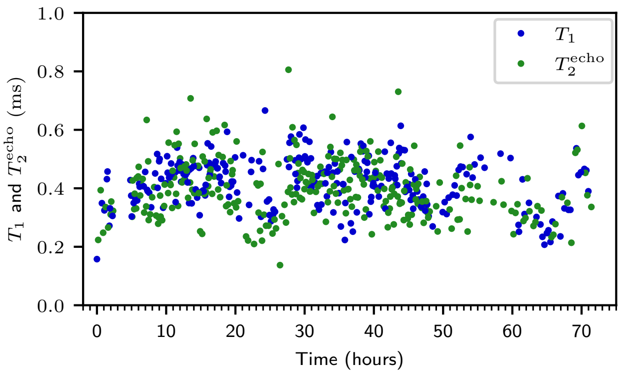

Supplementary Fig. 4. **Stability of the energy relaxation time T_1 and echo dephasing time T_2^{echo} measured during the second cooldown**. Energy relaxation and echo dephasing time as function of time over a 70-h period.

SUPPLEMENTARY NOTE 2: HIGH-COHERENCE TRANSMON QUBIT IN A 3D CAVITY

In this supplementary section, we present high-coherence transmon qubits coupled to three-dimensional (3D) cavity resonators to demonstrate the reproducibility of the fabrication process described in the main article. We measure two additional qubits Q_{S1} and Q_{S2} fabricated with an almost identical recipe as described in the main text. The only differences are the HF etching time which is 5 min instead of 10 min, and the junction oxidation pressure which is 1.5 mbar instead of 1.2 mbar. The qubits have an identical design, which is shown in Supplementary Fig. **5** and is available on Zenodo [1].

Qubits Q_{S1} and Q_{S2} are measured in 3D cavity resonators, C_{20} and C_{16}, respectively. Supplementary Fig. **6** presents illustrations of the cavities, which are made of 1050 aluminum alloy, and the design is available on Zenodo [1].

The qubits are measured in the same dilution refrigerator and with the same evaluation board as in the main article. Unlike in the main article, in this measurement setup, the readout signal and the qubit drive signal are combined into a single line by using a directional coupler, and we measure the reflection from the cavity instead of the transmission. The measurement setup is presented in the schematic diagram of Supplementary Fig. **7**, and the equipment is listed in Tables **3** and **4**. The cavity is protected from electromagnetic fields with a custom-designed four-layer magnetic shield. From the innermost to the outermost layer, the shield consists of 1.5-mm-thick gold-plated copper (C10100), 1.5-mm-thick aluminum 1050, and two layers of 1.5-mm-thick mumetal (Hymu80).

We measure that the cavities C_{20} and C_{16} have resonance frequencies of 5.55 GHz and 6.32 GHz, respectively. The frequency of qubit Q_{S1} is measured to be 2.9755 GHz and with an anharmonicity of -226 MHz. These parameters are 2.694 GHz and -230 MHz for the qubit Q_{S2}, respectively. We measure the qubit Q_{S1} to have a median energy relaxation time T_1 of 270 µs with the longest measured T_1 of (521 ± 27) µs. For the qubit Q_{S2}, the median T_1 is

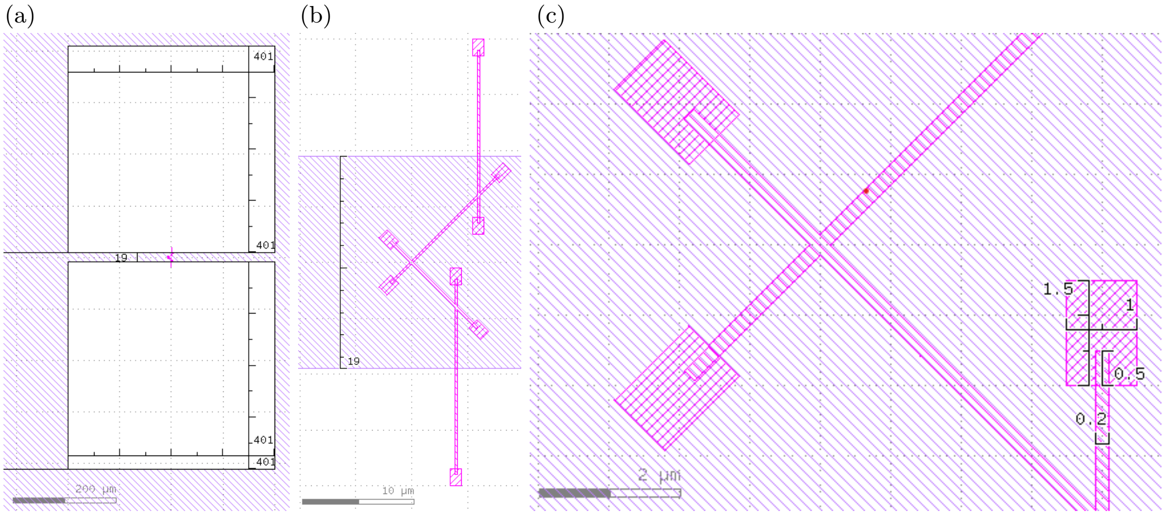

Supplementary Fig. 5. **Supplementary sample design.** (a) Design of the transmon samples used in the measurements of this Supplementary Note 1 and (b, c) magnifications in the vicinity of the Josephson junction. See full design file in Zenodo.

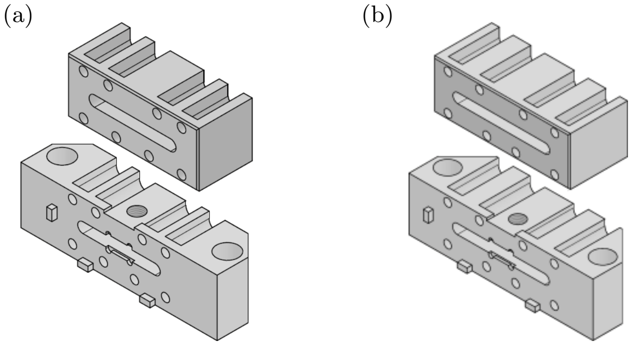

Supplementary Fig. 6. **Supplementary cavity design.** (a, b) Design illustrations of the 3D aluminum cavities (a) C_{16} and (b) C_{20} used in the experiments of this supplementary note. See full design file in Zenodo.

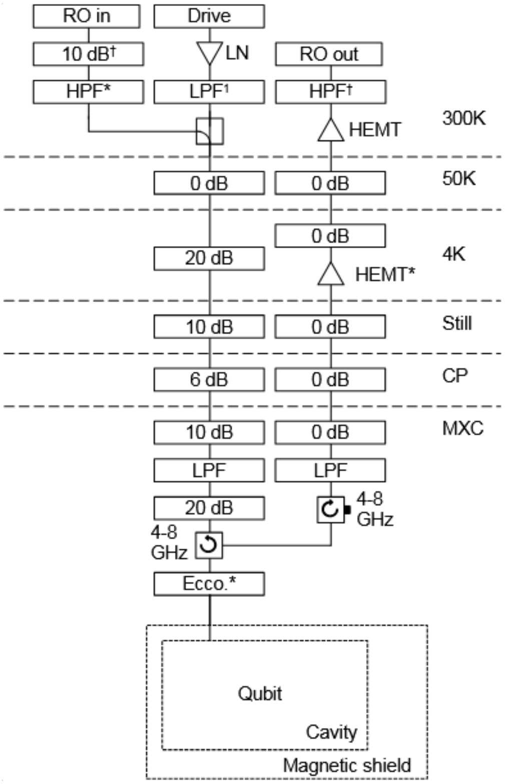

Supplementary Fig. 7. **Schematic of the measurement setup used in measurements of the qubits in cavities.** See Supplementary Table **3** and Supplementary Table **4** for a description of each component.

Supplementary Table 4. **Equipment used in the measurement setup of the supplementary note.**

Equipment	Description	Abbreviation
Low-pass filters	Mini-Circuits VLF-3400+ (3-dB point = 3.8 GHz)	LPF^1
Low-noise amplifier	Mini-Circuits ZX60-123LN-S+	LN
Directional coupler	Mini-Circuits ZUDC20-02183-S+	
High-pass filter	Mini-Circuits VHF-3100+ (20-dB stopband = DC–2.5 GHz)	HPF*
	Mini-Circuits VHF-4400+ (20-dB stopband = DC–3.85 GHz)	HPF†
Eccosorb filter	Kawashima Manufacturing 3.5mm Filter Connector L5 (0.32 dB/GHz)	Ecco.*
Circulator	Low Noise Factory LNF-CIC4_8A (4–8 GHz)	

measured to be 288 µs and the longest T_1 to be (496 ± 26) µs. Supplementary Fig. **8** presents distributions of the measured relaxation times and the time traces of the longest measured relaxation times. The data used to generate the figures are published on Zenodo [1].

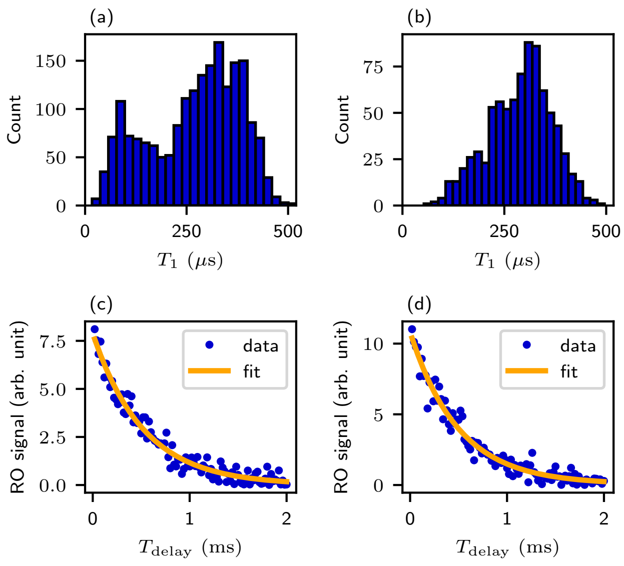

Supplementary Fig. 8. **Energy relaxation times of the additional qubits Q_{S1} and Q_{S2}.** (a, b) Distributions of the measured energy relaxation times of the qubits (a) Q_{S1} over 48 hours and (b) Q_{S2} over 40 hours. (c, d) Time traces for the longest measured relaxation times of (c) the qubit Q_{S1} with $T_1 = (521 \pm 27)$ µs and (d) the qubit Q_{S2} with $T_1 = (496 \pm 26)$ µs.

[1] M. Tuokkola, Y. Sunada, H. Kivijärvi, L. Grönberg, J.-P. Kaikkonen, V. Vesterinen, J. Govenius, and M. Möttönen, Data and GDS file for ”Methods to achieve near-millisecond energy relaxation and dephasing times for a superconducting transmon qubit (2024).
